# Cerebral cortex functional reorganization in preschool children with congenital sensorineural hearing loss: a resting-state fMRI study

**DOI:** 10.3389/fneur.2024.1423956

**Published:** 2024-06-25

**Authors:** Yi Yin, Xinyue Lyu, Jian Zhou, Kunlin Yu, Mingming Huang, Guiquan Shen, Cheng Hao, Zhengfu Wang, Hui Yu, Bo Gao

**Affiliations:** ^1^Department of Radiology, Affiliated Hospital of Guizhou Medical University, Guiyang, China; ^2^Guizhou Medical University, Guiyang, China; ^3^The Key Laboratory for Chemistry of Natural Product of Guizhou Province, Guizhou Medical University, Guiyang, China; ^4^Department of Radiology, Dermatology Hospital of Southern Medical University, Guangzhou, China; ^5^Key Laboratory of Brain Imaging, Guizhou Medical University, Guiyang, China

**Keywords:** congenital sensorineural hearing loss, cortex functional reorganization, functional MRI, functional connectivity, brain networks

## Abstract

**Purpose:**

How cortical functional reorganization occurs after hearing loss in preschool children with congenital sensorineural hearing loss (CSNHL) is poorly understood. Therefore, we used resting-state functional MRI (rs-fMRI) to explore the characteristics of cortical reorganization in these patents.

**Methods:**

Sixty-three preschool children with CSNHL and 32 healthy controls (HCs) were recruited, and the Categories of Auditory Performance (CAP) scores were determined at the 6-month follow-up after cochlear implantation (CI). First, rs-fMRI data were preprocessed, and amplitude of low-frequency fluctuation (ALFF) and regional homogeneity (ReHo) were calculated. Second, whole-brain functional connectivity (FC) analysis was performed using bilateral primary auditory cortex as seed points. Finally, Spearman correlation analysis was performed between the differential ALFF, ReHo and FC values and the CAP score.

**Results:**

ALFF analysis showed that preschool children with CSNHL had lower ALFF values in the bilateral prefrontal cortex and superior temporal gyrus than HCs, but higher ALFF values in the bilateral thalamus and calcarine gyrus. And correlation analysis showed that some abnormal brain regions were weak negatively correlated with CAP score (*p* < 0.05). The ReHo values in the bilateral superior temporal gyrus, part of the prefrontal cortex and left insular gyrus were lower, whereas ReHo values in the bilateral thalamus, right caudate nucleus and right precentral gyrus were higher, in children with CSNHL than HCs. However, there was no correlation between ReHo values and the CAP scores (*p* < 0.05). Using primary auditory cortex (PAC) as seed-based FC further analysis revealed enhanced FC in the visual cortex, proprioceptive cortex and motor cortex. And there were weak negative correlations between the FC values in the bilateral superior temporal gyrus, occipital lobe, left postcentral gyrus and right thalamus were weakly negatively correlated and the CAP score (*p* < 0.05).

**Conclusion:**

After auditory deprivation in preschool children with CSNHL, the local functions of auditory cortex, visual cortex, prefrontal cortex and somatic motor cortex are changed, and the prefrontal cortex plays a regulatory role in this process. There is functional reorganization or compensation between children’s hearing and these areas, which may not be conducive to auditory language recovery after CI in deaf children.

## 1 Introduction

Hearing loss is one of the most common sensory disorders in the world (1). Congenital sensorineural hearing loss (CSNHL) is a type of deafness that occurs before language development (2), and approximately 90% of hearing loss in affected children is moderate to severe. According to the World Health Organization, up to 1.5 billion people worldwide will suffer from some degree of hearing loss, and an additional 1.1 billion people are at risk of various types of hearing damage by 2050 (3). According to statistics, the proportion of permanent hearing loss caused by CSNHL is approximately 1–7‰ in newborns, and up to 30% of affected children have severe and/or extremely severe hearing loss (4). After hearing loss in children with CSNHL, due to the lack of sound stimulation, the development of the auditory center is affected to different degrees, and the auditory cortex will be repurposed in terms of function (5, 6). If children with CSNHL are deprived of sound stimulation for a long time, cerebral cortex function will change such that the auditory center will be requisitioned by other sensory modes (such as vision or proprioception) and cross-modal reorganization will occur (6), which will seriously affect the language acquisition of children with hearing loss and ultimately lead to intellectual development delays and cognitive function deficits in these children (7). In addition, this hearing loss and associated sequalae impose a heavy economic burden on families and society. Therefore, early diagnosis and early intervention are extremely important.

Currently, cochlear implantation (CI) is the most effective treatment for children with severe or extremely severe SNHL (8), with the purpose of partially or completely reversing the cross-modal reorganization of the auditory center, thus restoring the initial structure and function of the auditory cortex, with the goal of these children finally acquiring oral language communication abilities (9). The best time for CI is at 1 year of age, and CI should be performed in the auditory center sensitive period, between 1 and 3.5 years of age (10, 11), because this is the period when the greatest plasticity changes occur in the auditory system, and these changes can promote the maturity and development of the auditory cortex (12). However, some children still exhibit auditory and language behavioral impairments of varying degrees after CI surgery, which may be due to cortical functional reorganization or functional reorientation in the auditory center (13). At present, the P1 and N1 waveforms and the latency of cortical auditory evoked potentials and cortical visual evoked potentials are mainly used in clinical practice to determine whether cortical functional recombination has occurred (14, 15). These clinical evaluation methods are macroscopic and thus cannot be used to directly and effectively evaluate microscopic changes in cerebral cortical function in children with deafness before surgery. Therefore, it is very important to evaluate cerebral cortical function related to the auditory center in children with CSNHL early before CI, as cerebral cortical function serves as an important neuroimaging index for the clinical prediction of auditory recovery after CI.

Resting-state functional MRI (rs-fMRI) (16) is an advanced non-invasive magnetic resonance imaging technique that assesses regional brain activity and functional connectivity by measuring low-frequency spontaneous blood oxygen level-dependent signals, and low-frequency fluctuation (ALFF), regional homogeneity (ReHo) and functional connectivity (FC) are good neural markers for brain imaging. Therefore, researchers have used rs-fMRI and these neural markers to explore the reorganization of cerebral cortex in deaf patients (17–19). For example Wang et al. (20) found that children with CSNHL had abnormal changes in ReHo values in local brain regions, indicating that deafness had an impact on brain functional activity in children. Guo et al. (21) found brain functional alterations as indicated by rs-fMRI ReHo values in the brain regions related to auditory, visual, motor, and cognitive function in children with CSNHL. In addition, a study (22) showed that sensorineural hearing loss (SNHL) patients displayed abnormal dALFF value in related visual cortices, and these findings suggest that SSNHL patients experience cross-modal plasticity and visual compensation, which may be closely related to the pathophysiology of SNHL. For FC studies, in children with severe SNHL without cochlear implants, the primary auditory cortex was found to have fewer FC to the motor cortex, while the visual cortex had more FC to the motor cortex and speech cortex (23). Liu et al. evaluated whole-brain FC changes related to the auditory cortex in adult patients with left-sided SNHL and found these patients exhibited striking FC changes in the auditory system, recognition network, visual cortex and language network (24). These findings suggest that cortical functional recombination is an intrinsic adaptation or compensatory change in the multiple senses of the brain to the loss of auditory cortical function and is a regulatory mechanism in the functional regulation circuit of the brain (25). This information is helpful for fully understanding the cortical recombination mechanism of the auditory cortex center and its related functional areas after hearing loss and provides a reliable imaging basis for the preoperative evaluation of CI. In recent years, it was found that the prefrontal cortex (PFC) (26) plays a top-down regulatory role in the auditory pathway and can mediate and regulate the reorganization of the auditory cortex, visual cortex, and proprioceptive cortex (27). However, the mechanism underlying PFC-mediated cortical functional recombination of these cortical regions after auditory deprivation remains unclear. Most of these previous studies have focused on older deaf patients with longer periods of auditory deprivation who have matured beyond the sensitive period of hearing development and plasticity. However, there are few reports on fMRI studies of cerebral cortical functional recombination in preschool children with SNHL, leaving gaps in knowledge.

In summary, most of the research on the use of rs-fMRI in deaf patients has involved children or adults, but few studies have been reported in preschool children with CSNHL, and the mechanisms underlying cerebral cortical functional recombination after auditory deprivation in deaf children are still unclear. Therefore, rs-fMRI was used to directly analyze the functional recombination changes in the auditory cortex of preschool children with double-focus SNHL and the correlation between functional changes in brain regions and clinical Categories of Auditory Performance (CAP) scores in this study. The purpose of this study was to better understand the brain FC characteristics associated with preschool CSNHL and to provide a useful neuroimaging basis for predicting auditory rehabilitation in preschool children with severe SNHL after CI.

## 2 Materials and methods

### 2.1 Patients

Initially, 70 children with CSNHL, ranging in age from 0 to 6 years, who were to undergo CI surgery between May 2017 and June 2020 at the Affiliated Hospital of Guizhou Medical University were recruited for the present study. At the same time, 38 healthy controls (HCs) with normal hearing were recruited from the hospital. All CSNHL participants were subjected to hearing screening. All the patients with auditory brain response (ABR) results greater than 90 dB were documented as having bilateral profound hearing loss. The patients were then referred by the Department of Otolaryngology for MRI scans with sedation as a presurgical evaluation for CI, and the patients’ parents provided consent for their children to participate in our fMRI protocol. Any patient who had any malformation or abnormality found in the high-resolution computed tomography scan (HRCT) of the temporal bone or MRI scan of the brain and inner ear was not included in the study. Deaf infant participants who used hearing aids or had a history of infections, ototoxic drug use, cytomegalovirus, trauma, or any other neurological disease were excluded from the study. The HCs group was well matched to the patient group in terms of age and sex. Participants in the HC group underwent clinical MRI scans with sedation for nonhearing-related indications, and their parents agreed to additional sequence scans and hearing tests. The HCs group inclusion criteria were age ranging from 0 to 6 years, no frequency greater than 25 dB and normal neuroanatomy as determined by a pediatric neuroradiologist. The HCs group exclusion criteria were a diagnosis of various central nervous system diseases, such as white matter hypoplasia, abnormal neuronal migration, trauma, tumors, infection, and epilepsy. All participants’ parents signed informed consent forms prior to their child entering the study. This study was approved by the Ethics Committee of the Affiliated Hospital of Guizhou Medical University.

Clinical score 6 months after CI: Sixty-three preschool children with CSNHL underwent CI (all CI devices were the same brand and were provided by the same manufacturer). No related complications were observed in this study. Children who underwent CI returned to our hospital one month after the operation for startup and debugging and returned again six months after startup for CAP testing. The scoring standard refers to the CAP proposed by Nikolopoulos et al. (28). CAP is divided into 10 grades (see Supplementary Table S6), which mainly reflect the real hearing level of patients in life.

### 2.2 MRI acquisition

In this study, we used a Philips Achieva 3.0 T MR scanner with an 8-channel head coil to obtain MR images for all participants before they received any treatment. Due to the relatively young age of the participants in this study, 10% chloral hydrate solution was administered orally to the deaf children 15 min before MRI examination at a dose of 50–60 mL/kg to ensure MRI scan quality. Earplugs and headphones were provided for hearing protection. During MRI, infants’ oxygen saturation was continuously monitored by a pulse oximeter, and infants were closely observed by a pediatrician. All infants underwent anatomical MRI and fMRI acquisitions using the protocol detailed below.

Anatomical images, including higher solution T1-weighted images, were acquired by using a three-dimensional brain volume (3D-BRAVO) sequence with the following parameters: echo time (TE) = 4.6 ms, repetition time (TR) = 9.2 ms, flip angle (FA) = 8°, slice thickness = 1.6 mm, slice interval = 0.8 mm, field of view (FOV) = 220 × 220 mm^2^, acquisition matrix = 276 × 227, number of slices =180, and scanning time = 5 min 24 s.

Rs-fMRI data were acquired using the echo-planar imaging sequence, with the following parameters: TE = 30 ms, TR = 2000 ms, time point = 200, FA = 90°, FOV = 220 × 220 mm2, slice thickness = 3.40 mm, number of slices = 35, and scanning time = 6 min 46 s.

### 2.3 MRI data processing and quality control

Resting-state fMRI data were preprocessed using Data Processing and Analysis for Brain Imaging (DPABI) (29) and Statistical Parametric Mapping (SPM12) software^1^ in the MATLAB R2020a platform. First, we converted the files from the DICOM format into a standard NIFTI format. Second, the first 10 time points in the series of rs-fMRI data were discarded to avoid errors caused by unstable magnetic fields. Third, slice time correction, head motion correction, and covariate removal were performed on the rs-fMRI data. Fourth, the initial coordinates of the T1-weighted images (T1WIs) and fMRI images were manually located to the anterior commissure, and images with excessive displacement and rotation deviations were corrected manually. Fifth, the processed images were normalized to Montreal Neurological Institute (MNI) space. Finally, the images were filtered, bandpass filtered (0.01–0.08 Hz), and smoothed with a 6 mm full width at half maximum (FWHM) isotropic Gaussian kernel. Any image with head motion >2 mm translation or 2° rotation in any direction was excluded.

For rs-fMRI data, 3 CSNHL patients with head motion (translation >2 mm and/or rotation >2°) were excluded from further analysis. In addition, the author visually inspected the coregistration and normalization in fMRI data processing in all datasets. Structural and functional image coregistration failed for 4 CSNHL participants, while brain template normalization failed for 3 HCs. Thus, 7 CSNHL patients and 3 HCs were excluded from further analyses. Ultimately, 63 children with CSNHL and 32 HCs were eligible for this study.

### 2.4 Amplitude of low-frequency fluctuation and regional homogeneity analyses

All images were normalized to the standard space on the Montreal Neurological Institute (MNI) template and were resampled to 3 × 3 × 3 mm^3^. The MNI template has been known to be appropriate for normalizing brains from young children to adolescence (30). Subsequently, linear trend, white matter signal, cerebrospinal fluid signal, and Friston 24 motion parameters were used as regressors to reduce effects of head movement and non-neuronal information (31).

The ALFF values can indicate the level of spontaneous activity of single voxel brain in resting state. ALFF values were calculated using rs-fMRI (DPABISF) (32). The time series of each voxel was transformed to the frequency domain using fast Fourier transform (FFT) (parameters: taper percent = 0, FFT length = shortest), and the power spectrum was obtained. Then, the power spectrum obtained by FFT was square rooted and averaged across 0.01 to 0.08 Hz at each voxel. This averaged square root was taken as the ALFF values. In order to improve the normality of the results and reduce the impact of differences between individuals on the results, the ALFF value was further normalized, that is the average value of the whole brain ALFF was subtracted and divided by its standard deviation to obtain the normalized zALFF value. Finally, all images were further smoothed by a Gaussian kernel with a FWHM of 6 mm. And the signal-to-noise ratio of all images is improved and the residual of all images conforms to the Gaussian distribution.

The ReHo brain map was generated by calculating Kendall’s coefficient of concordance (KCC) between each voxel and its 26 nearest neighboring voxels using unsmoothed data (0.01–0.08 Hz). Then, to eliminate the effect of individual diversification, the KCC-ReHo value was normalized to the KCC-ReHo z value. A Gaussian kernel of 6 mm FWHM was used to spatially smooth the standardized ReHo maps (33).

### 2.5 Seed-based resting-state FC analysis

Resting-state FC analysis was performed using the seed-voxel correlation approach, in which the time-course signal in a seed region is correlated to all voxels in the whole brain. For a particular A1 seed, a mask of the left and right PAC (34) was defined as a region of interest (ROI) based on automated anatomical labeling (AAL) (35). Correlation coefficients were then transformed to z values using the Fisher r-to-z transformation to increase normality.

### 2.6 Statistical analysis

The two-sample *t*-test (two-tailed, *p* < 0.05) was used to assess between-group differences with respect to demographic and clinical characteristics (age, sex, and ABR) using SPSS 25.0 software (IBM, Armonk, NY, United States). Voxel-wise two-sample *t*-test was implemented to match the differences in the variance ALFF ReHo and FC values between the preschool children with CSNHL and HC, using sex age and head motion parameters as covariates, with the software DPABI^2^ (32). The variances of ALFF ReHo and FC were corrected by the false discovery rate (FDR, at the voxel level *p* < 0.005 and at the cluster level *p* < 0.05) with software SPM8.^3^ Finally, Pearson’s correlation analysis was applied to test the relationship between neuroimaging parameters (ALFF, ReHo and FC value) and clinical CAP score of preschool children with CSNHL.

## 3 Results

### 3.1 Demographic and clinical characteristics

The demographic and clinical data of 63 preschool children with CSNHL (33 males and 30 females, mean 3.40 ± 1.41 years old) and 32 HCs (17 males and 15 females, mean 3.16 ± 1.73 years old) are shown in Table 1. No significant differences were found in age or sex between the groups. The ABRs of the left and right ears were significantly different between the groups.

**Table 1:** Demographic and clinical data of all participants.

Characteristics	Group: mean *±* SD		*c*^2^/*t*	*p*-value
	CSNHL (*n* = 63)	HCs (*n* = 32)		
Sex (male/ female) ^a^	33/30	17/15	*c*^2^ = 0.0047	0.9453
Age(years)^b^ (mean *±* SD)	3.70 ± 0.18	3.16 ± 0.73	*t* = 1.6050	0.1120
Age range (years)	0.11–6.60	0.80–6.00	-	-
Right ear ABR(dB nHL)^b^ (mean *±* SD)	93.57 ± 1.32	15.25 ± 0.81	*t* = 50.390	<0.0001

All continuous data are presented as mean ± SD; ^a^Chi-square tests; ^b^Unpaired Student’s *t*-tests. CSNHL, congenital sensorineural hearing loss; HCs, healthy controls; ABR, auditory brainstem response; SD, standard deviation.

### 3.2 ALFF and ReHo analyses

**Figure 1:**
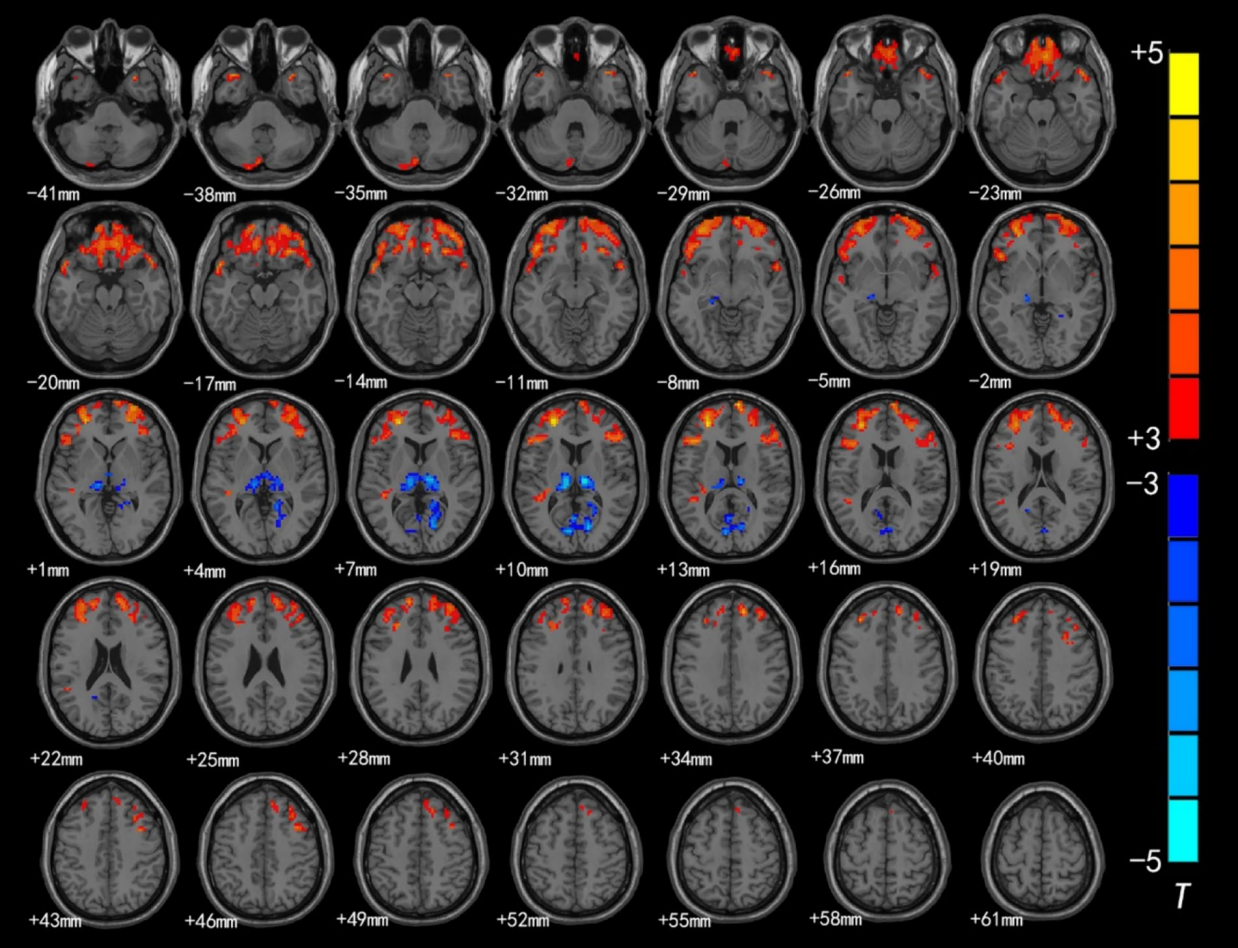
Brain region map showing significant differences in ALFF between the CSNHL group and the HC group. The areas with decreased ALFF values were the bilateral superior frontal gyrus (BA9), bilateral middle frontal gyrus (BA10), left inferior frontal gyrus (BA47), bilateral orbitofrontal gyrus (BA11) and bilateral superior temporal gyrus (BA38); the areas with increased mALFF values were the bilateral thalamus and calcarine fissure cortex (BA18) (FDR corrected, *p* < 0.05). The color bar represents the *T* values.

Brain regions with significant differences in ALFF values between preschool children with CSNHL and HCs are presented in Table 2 and Figure 1. Two-sample *t*-tests showed that the ALFF values in the bilateral superior frontal gyrus (BA9), bilateral middle frontal gyrus (BA10), left inferior frontal gyrus (BA47) and bilateral orbitofrontal gyrus (BA11) of the CSHNL group were significantly lower than those of the HCs group (*p* < 0.05, FDR correction). In addition, the ALFF value in the bilateral thalamus and calcarine gyrus (BA17) of the CSNHL group was higher than that of the HCs group (*p* < 0.05, FDR correction).

**Table 2:** Brain regions with ALFF differences between preschool children with CSNHL and HCs.

Brain regions	BA	MNI coordinates (mm)			Cluster size (mm^3^)	*T* value
		x	y	z		
**HCs > CSNHL**						
Left medial frontal gyrus	10	−24	+48	+9	261	7.78
Right medial frontal gyrus	10	+27	+54	+0	234	5.13
Right superior frontal gyrus	9	+15	+57	+21	148	4.91
Left superior frontal gyrus	9	−18	+54	+18	136	4.78
Left orbitofrontal gyrus	11	−9	+36	−21	146	5.18
Right orbitofrontal gyrus	11	+12	+39	−21	96	4.73
Right superior temporal gyrus	38	+51	+15	−21	62	4.44
Left superior temporal gyrus	38	−51	+15	−18	48	4.19
**HCs < CSNHL**						
Right thalamus	-	+12	−18	+9	83	−4.92
Left thalamus	-	−9	−18	+6	95	−4.04
Right calcarine gyrus	17	+15	−72	+9	77	−4.52
Left calcarine gyrus	17	−6	−77	+9	46	−4.34

MNI, Montreal Neurological Institute; CSNHL, congenital sensorineural hearing loss; HCs, healthy controls.

**Figure 2:**
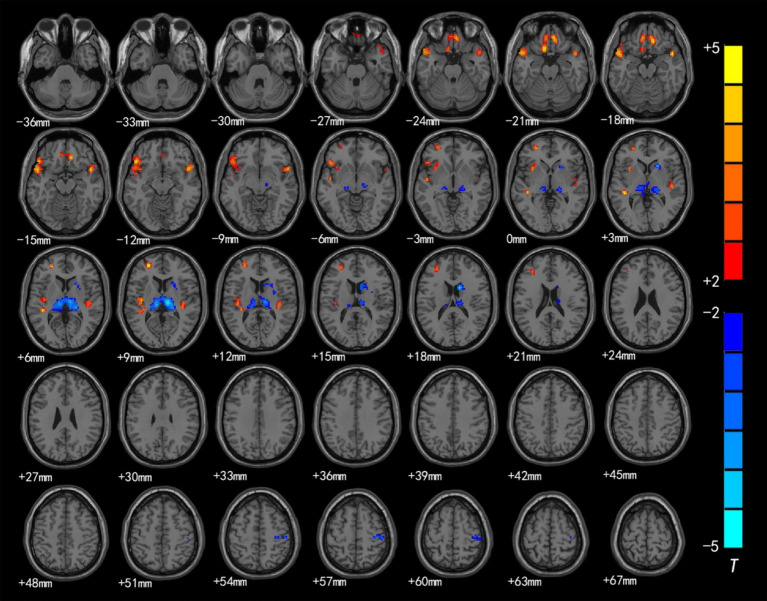
Brain region map showing significant differences in ReHo between the CSNHL group and the HC group. The areas with decreased ReHo were the bilateral superior temporal gyrus, bilateral orbitofrontal gyrus, left middle frontal gyrus, left inferior frontal gyrus (BA47) and left insula; the areas with increased ReHo included the bilateral thalamus, right caudate nucleus, and right anterior central gyrus (FDR corrected, *p* < 0.05). The color bar represents the *T* values.

The brain regions with significant differences in ReHo values between preschool children with CSNHL and HCs are presented in Table 3 and Figure 2. Two-sample *t*-tests showed that the CSNHL group had higher ReHo values in the bilateral superior temporal gyrus (BA38, 41), bilateral orbitofrontal gyrus (BA11), left medial frontal gyrus (BA10), left inferior frontal gyrus (BA47) and left insula (BA13) than the HCs group. In addition, the ReHo value in the bilateral thalamus, right caudate nucleus and right anterior central gyrus (BA3) of the CSNHL group was higher than that of the HCs group (*p* < 0.05, FDR correction).

**Table 3:** Brain regions with ReHo differences between preschool children with CSNHL and HCs.

Brain regions	BA	MNI coordinates (mm)			Cluster size (mm^3^)	*T* value
		x	y	z		
*HCs > CSNHL*						
Right superior temporal gyrus	38	48	12	−15	77	5.28
Left superior temporal gyrus	38	−54	12	−21	93	5.01
Left orbitofrontal gyrus	47	−48	27	−12	92	5.10
Right orbitofrontal gyrus	11	12	33	−18	65	5.27
Left medial frontal gyrus	10	−24	48	9	60	5.16
Left superior temporal gyrus	41	−42	−33	3	30	5.32
Left insula	13	−30	18	0	39	3.53
*HCs < CSNHL*						
Right thalamus	-	9	−18	9	168	−6.14
Left thalamus	-	−9	−21	9	138	−5.15
Right caudate nucleus	-	12	9	18	43	−5.73
Right anterior central gyrus	3	45	−21	57	29	−3.94

MNI, Montreal Neurological Institute; CSNHL, congenital sensorineural hearing loss; HCs, healthy controls.

### 3.3 Seed-based resting-state FC analysis

We selected left/right PAC based on the AAL atlas as the seed and used this seed in whole-voxel FC analysis.

**Figure 3:**
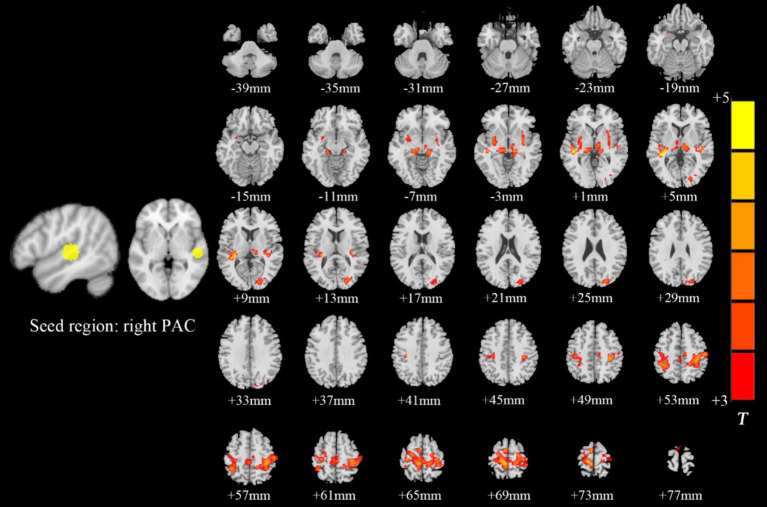
Brain map of FC with the right PAC as the seed point. FC was enhanced between the right PAC and the bilateral superior temporal gyrus, left superior frontal gyrus, left paracentral lobule, right posterior central gyrus, right occipital lobe, bilateral thalamus, and bilateral insulas. Results after FDR correction are shown; *p* < 0.05. The color bar represents the *T* values.

**Figure 4:**
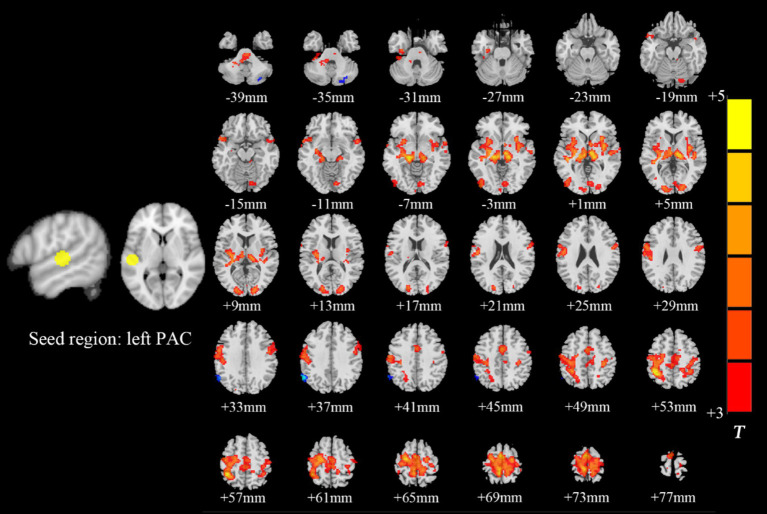
Brain map of FC with the left PAC as the seed point. FC was enhanced between the left PAC and the bilateral anterior central gyrus, posterior central gyrus, left inferior parietal lobule, bilateral occipital lobe, dorsal anterior cingulate cortex, bilateral thalamus, bilateral insula and right superior temporal gyrus. Results after FDR correction are shown; *p* < 0.05. The color bar represents the *T* values.

Intragroup comparisons revealed extensive positive FC with the right PAC in both groups (Figure 3; Table 4). These areas included the bilateral superior temporal gyrus, left superior frontal gyrus, left paracentral lobule, right posterior central gyrus, right occipital lobe, bilateral thalamus, and bilateral insula. When we used the left PAC as the seed point for FC analysis, the FC between the left PAC and the bilateral anterior central gyrus, posterior central gyrus, left inferior parietal lobule, bilateral occipital lobe, dorsal anterior cingulate gyrus, bilateral thalamus, bilateral insula, and right superior temporal gyrus was enhanced (Figure 4; Table 5). Most of these FC-enhanced brain regions are considered part of the auditory network, sensorimotor network, sensorimotor network, and default mode network (DMN).

**Table 4:** Brain regions with differences in right seed-based FC analysis.

Seeds	Brain regions	BA	MNI coordinates (mm)			Cluster size (mm^3^)	*T* value
			x	y	z		
**Right PAC**							
	Right posterior central gyrus	4	33	−33	51	349	4.58
	Left paracentral lobule	4	−9	−33	69	172	4.93
	Left superior temporal gyrus	41	−39	−36	6	64	5.27
	Right superior temporal gyrus	41	48	−21	3	31	4.48
	Left superior frontal gyrus	6	−6	−6	69	62	4.48
	Right cuneiform lobe	17	21	−84	12	42	3.96
	Right supraspinal gyrus	19	24	−84	27	77	4.12
	Right thalamus	-	12	−15	3	79	3.87
	Left thalamus	-	−15	−24	0	58	4.12
	Right insula	-	−33	−9	−3	58	4.33
	Left insula	-	−30	−12	0	41	3.59
	Left parahippocampal gyrus	30	−15	−27	−9	42	4.27

MNI, Montreal Neurological Institute; FC, functional connectivity; PAC, primary auditory cortex.

**Table 5:** Brain regions with differences in left seed-based FC analysis.

Seeds	Brain regions	BA	MNI coordinates (mm)			Cluster size (mm^3^)	*T* value
			x	y	z		
*Left PAC*							
	Left anterior central gyrus	6	−24	−12	63	306	5.21
	Left posterior central gyrus	4	−6	−39	69	364	4.36
	Left inferior parietal lobule	40	−39	−45	54	153	5.68
	Right posterior central gyrus	4	12	−36	69	171	4.28
	Left thalamus	-	−17	−27	−6	125	5.60
	Right thalamus	-	9	−18	0	121	5.22
	Right anterior central gyrus	6	54	0	27	95	4.17
	Left precuneus	17	−9	−44	68	61	4.38
	Left superior occipital gyrus	19	−18	−84	15	49	4.25
	Right superior occipital gyrus	18	21	−87	9	32	4.19
	Middle cingulate gyrus	24	0	3	45	39	4.32
	Right insula	48	36	−3	3	48	4.79
	Left insula	48	−30	−12	3	65	3.68
	Right superior temporal gyrus	41	48	−21	3	47	4.65

MNI, Montreal Neurological Institute; FC, functional connectivity; PAC, primary auditory cortex.

### 3.4 Correlation analysis of ALFF, ReHo and FC values with CAP scores

#### 3.4.1 Correlation analysis of ALFF and ReHo

**Figure 5:**
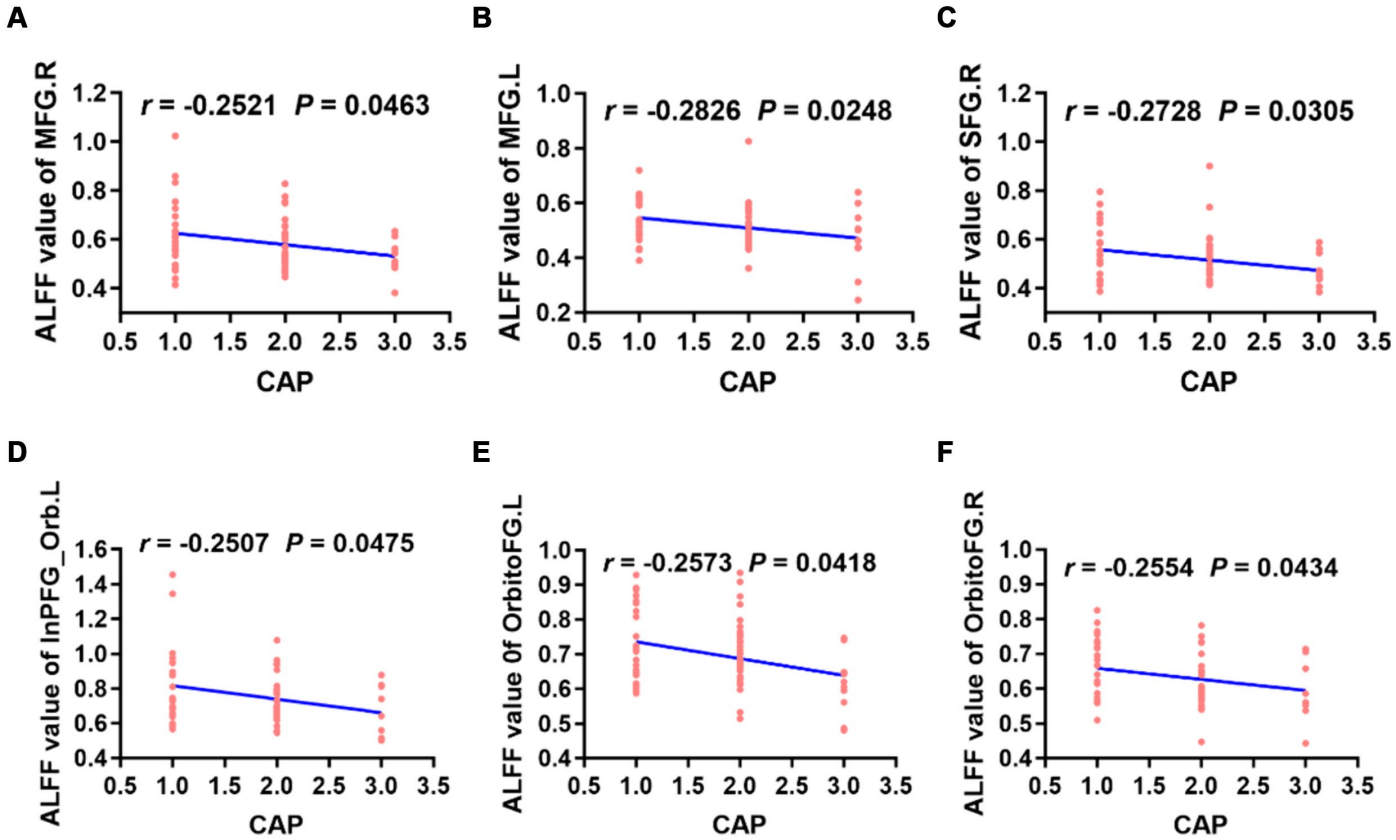
Correlation analysis revealed that the ALFF values of the bilateral MFG, right SFG, left InPFG_Orb and bilateral OrbitoFG were weakly negatively correlated with CAP scores in preschool children with CSNHL **(A–F)**. MFG, middle frontal gyrus; InPFG, inferior prefrontal gyrus; Orb, orbital; OrbitoFG, orbital frontal gyrus; CSNHL, congenital sensorineural hearing loss; CAP, categories of auditory performance.

The ALFF values in the left/right middle frontal gyrus, right superior frontal gyrus, left inferior frontal gyrus, right orbitofrontal gyrus and left orbitofrontal gyrus were weakly negatively correlated with CAP scores (Figure 5). However, there was no correlation between the ReHo value in each brain region and the CAP score.

#### 3.4.2 Correlation analysis based on seed FC values

**Figure 6:**
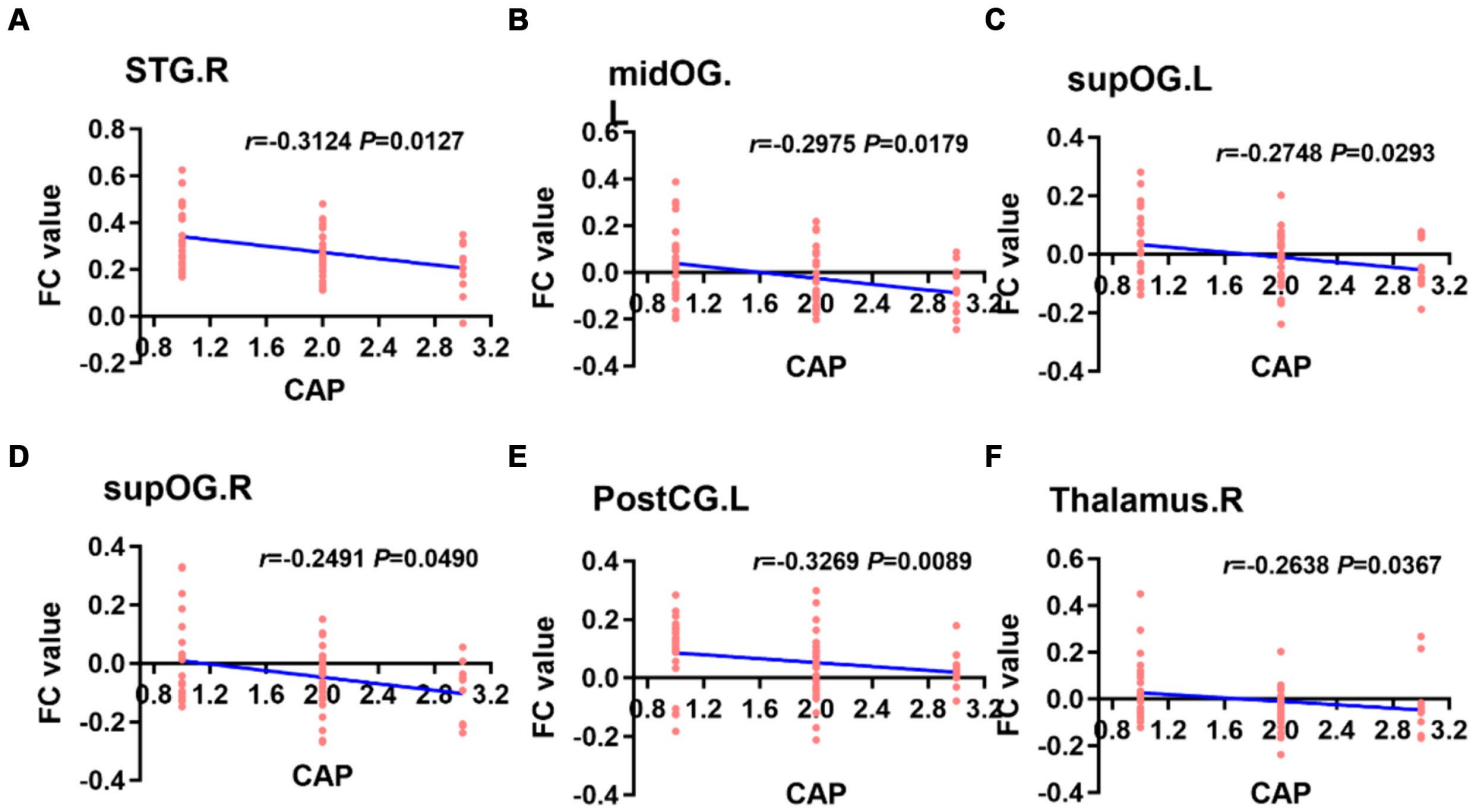
Based on the left PAC seed analysis, the brain regions with a weakly negatively correlation between the FC value and the CAP score were the right STG, left midOG, bilateral supOG, PostCG and right side of the thalamus **(A–F)**. STG, superior temporal gyrus; midOG, middle occipital gyrus; supOG, superior occipital gyrus; PostCG, posterior central gyrus; CAP, categories of auditory performance.

**Figure 7:**
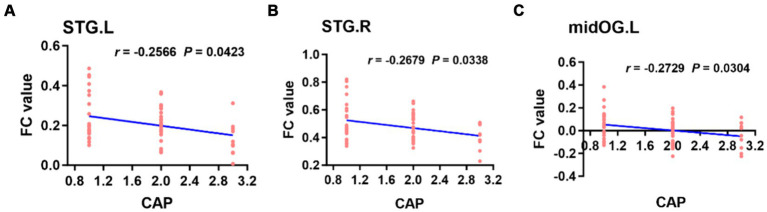
Based on the right PAC seed analysis, the brain regions in which the FC value was weakly negatively correlated with the CAP score in the bilateral STG of the auditory region and left midOG **(A–C)**. STG, superior temporal gyrus; midOG, middle occipital gyrus; CAP, categories of auditory performance.

When the left PAC was used as the seed, the FC values of the right superior temporal gyrus, left occipital lobe, right occipital lobe, left posterior central gyrus and right thalamus were weakly negatively correlated with the CAP score (Figure 6). When the right PAC was used as the seed, the FC values of the left superior temporal gyrus, right superior temporal gyrus and left occipital lobe were weakly negatively correlated with the CAP scores (Figure 7).

## 4 Discussion

In recent years, with the continuous development of advanced noninvasive MRI technology for assessing cognitive function and neuropsychological and emotional regulation, rs-fMRI has been applied to study the underlying neuropathological mechanisms of SNHL (21, 34).

In this study, rs-fMRI data were obtained from preschool children with CSNHL. First, ALFF and ReHo analyses were performed to analyze changes in local brain function in children with SNHL after auditory deprivation. Seed-based FC analyses were subsequently performed to further explore the changes in the bilateral PAC and whole-brain functional network connectivity in deaf children. Finally, the correlation between ALFF, ReHo and FC values in different brain regions and CAP scores were calculated. The results of these analyses showed that the local functions of the auditory cortex, visual cortex and sensorimotor cortex of the brain were altered and that cortical functions were reorganized overall after auditory deprivation in preschool children with CSNHL. The reorganization of these cortical functions may be detrimental to hearing and speech recovery after CI surgery. We hope that our findings will complement those of previous studies and help to better explain the underlying pathophysiological mechanisms of CSNHL in preschool children.

### 4.1 Changes in ALFF and ReHo in terms of local brain function in preschool children with CSNHL

ALFF is defined as the low-frequency vibration amplitude of the brain BOLD signal in the low-frequency range (0.01–0.08 Hz), reflecting the spontaneous activity of local neurons in the cerebral cortex (21, 36). An increase or decrease in ALFF reflects abnormal metabolic activity of local neurons (36). The ReHo map can reflect the similarity or synchronicity of low-frequency brain vibration signal fluctuations in the region (37). Abnormal ReHo activity indicates local brain desynchronization (38). Therefore, ALFF and ReHo can be used as indicators of neurophysiological changes in different brain diseases.

#### 4.1.1 Changes in ALFF and ReHo in the auditory cortex, visual cortex and somatomotor cortex of preschool children with CSNHL

In this study, we observed that ALFF and ReHo were significantly decreased in the bilateral superior temporal gyrus and that there was a decrease in ReHo in the left insula among individuals with SNHL. Furthermore, there was a significant increase in ALFF in the bilateral calcarine gyrus and an increase in ReHo in the right anterior central gyrus.

The auditory speech center comprises the PAC, secondary auditory cortex, and auditory association cortex. The PAC is located in BA41, while the secondary auditory cortex is situated in BA22 and BA42. The PAC resides within the superior temporal gyrus (Heschl’s gyrus) (39), which is primarily responsible for auditory perception and coreceiving sound stimulation alongside the secondary auditory cortex. However, the functions of the secondary auditory cortex are more intricate, encompassing hearing, language processing, and executive attention, and the secondary auditory cortex exhibits extensive connections with other brain regions involved in audition (such as the PFC and parietal lobe) (40). The present study revealed a significant reduction in ALFF and ReHo in the PAC among children with SNHL. This finding suggested that the auditory cortex is impacted following hearing loss in deaf children, potentially due to functional reorganization or cross-modal integration. The development of the auditory nervous system involves rapid growth during the critical or sensitive period. Insufficient sensory stimulation, such as hearing, during this time can have varying degrees of impact on the development and functioning of the auditory center, ultimately leading to the repurposing of the auditory cortex (5). Prolonged auditory deprivation may result in cross-modal reorganization, where other sensory modes, such as vision or proprioception, take over the requisitioned auditory center and elicit responses to different sensory stimuli (6, 13, 41). This theory could explain the decreased activity of the bilateral superior temporal gyrus in children with SNHL. In addition, the ReHo of the left insula in children with SNHL was decreased in the present study because the insula is an important node in the auditory network, which is mainly responsible for the recognition of music, cries and laughter (42) and is involved in advanced cognitive functions such as auditory attention and memory (43). Therefore, the activity of the left insula is reduced in deaf children, suggesting that the loss of auditory cortex function may affect the further processing of auditory stimuli by the insula.

In BA17, the calcarine gyrus is the gyrus located between the upper and lower parts of the calcarine sulcus, which is the site where the PAC is concentrated and can process visual information (44). Therefore, the calcarine gyrus belongs to the visual-related cortex and is mainly involved in visual processing. Studies have shown that deaf people have better visual performance than hearing people (45). In addition, some studies have confirmed that patients with deafness usually have better visual function in the task of attention concentration, and this better visual function is more obvious in patients with congenital prelingual deafness (46, 47). Xia and colleagues (17) showed that, compared with HCs, children under 4 years of age with severe SNHL had significantly reduced ALFF and ReHo in the auditory and language-related cortices but increased ALFF and ReHo in the right occipital cortex (BA18, BA19) according to rs-fMRI. These studies suggest a cross-modal reorganization or visual cortex function compensation after auditory deprivation in deaf patients. The results of this study showed that the ALFF and ReHo of the superior temporal gyrus were significantly decreased in preschool children with CSNHL, while the ALFF of the visual area was increased, which also demonstrated visual cortex function compensation or cross-modal reorganization after auditory deprivation in deaf children.

The precentral gyrus, which contains many giant pyramidal cells, is the center of body movement in humans and an important structure for executing voluntary movements (48). Previous studies have confirmed that in infants, language ability is acquired by learning the connection between pronunciation and movements through sound stimulation (49), which indicates that the synchronous coordination of functions between the auditory cortex and the motor cortex may be an important factor in language development (50). Recent studies have shown that children with severe SNHL have no significant changes in the visual cortex, but there are significant changes in connections between the auditory cortex and the somatosensory and sensorimotor cortices (23). In this study, preschool children with CSNHL had increased ALFF and ReHo in the right precentral gyrus, suggesting that the functional activity of the somatosensory cortex was increased after auditory deprivation, which may be the result of reorganization or compensation for the reduced activity of the auditory cortex by the somatosensory cortex.

In conclusion, preschool children with CSNHL have reduced auditory central cortex function but increased visual cortex and somatomotor cortex function, indicating that reorganization or compensation by the visual and somatomotor cortex occurs after auditory deprivation.

#### 4.1.2 Changes in ALFF and ReHo in the PFC of preschool children with CSNHL

In this study, we found that ALFF and ReHo were decreased in the bilateral orbitofrontal gyrus and left inferior frontal gyrus (BA47) in SNHL patients, while ALFF was significantly decreased in the bilateral superior frontal gyrus, and ReHo was significantly decreased in the left middle frontal gyrus. The bilateral superior frontal gyrus, middle frontal gyrus, orbitofrontal gyrus and inferior frontal gyrus (BA47) belong to the PFC (51). Some studies have shown that the PFC has a top-down regulatory effect on sensory (auditory and visual) perception through the prefrontal-temporal pathway (40, 52). Such regulation can be close-distance regulation or long-distance regulation across regions. Anatomically, the dorsolateral PFC has a close anatomical connection with the auditory pathway and plays an important role in multisensory (such as hearing and vision) integration and processing (51, 53). Studies have confirmed that the PFC has an early inhibitory effect on PAC input in humans and that the PFC is related to auditory attention, with a top-down regulatory effect on auditory processing (26). The tight connectivity between the PFC and sensory areas (auditory, visual or proprioceptive) provides a convenient circuit for cortical functional reorganization (54). Thus, in cases of sensory deprivation, cross-modal reorganization is also mediated by the attentional transition from the deprived modality to the retained modalities via top-down modulation (54). The takeover of prefrontal high-order cognitive areas in the auditory cortex might thus be a major pattern of functional reorganization in SNHL (55). In the present study, the ALFF of the superior frontal gyrus, middle frontal gyrus, and left inferior frontal gyrus was significantly reduced in children with CSNHL, suggesting that the local neuronal activity in these regions was reduced, which may be caused by PFC-mediated cortical reorganization.

### 4.2 Changes in whole-brain FC in preschool children with CSNHL

FC was analyzed based on the temporal correlation between each brain area and the brain functional network. These brain areas have similar functional characteristics to the related functional network (56, 57), and the synchronization or consistency of neuronal activity is reflected by the synchronous BOLD activity. Increased FC indicates activity synchronization and functional correlation between two voxels or brain regions (58).

#### 4.2.1 FC changes in the auditory and visual cortices in preschool children with CSNHL

In this study, seed-based FC analysis revealed that preschool children with CSNHL had significantly increased FC in the bilateral superior temporal gyrus (BA41), occipital lobe (BA18, 19) and insular lobe. The superior temporal gyrus (BA41) is in the PAC, and its main functions are to receive and perceive sound stimuli (39, 59). The occipital lobe (BA18, 19), as the center of the primary, intermediate and higher visual cortices, is the main site of retinal signal input and is responsible for processing visual information (44, 60). After long-term hearing loss in deaf patients, different degrees of functional activation occur in the auditory cortex and nonauditory cortex regions (such as the visual cortex, motor cortex and proprioceptive cortex) of deaf patients, which indicates a functional reorganization of the auditory cortex after auditory deprivation (40). In our study, we first performed ALFF and ReHo analyses to study preschool children with CSNHL, and the results confirmed that the activity of the auditory area was reduced while the activity of the visual center was significantly increased in children with deafness. Then, we performed seed-based FC analysis to show that the FC of auditory and visual areas was significantly enhanced, which fully proved that cross-modal reorganization of cortical function occurred in the visual cortex and auditory cortex after auditory deprivation in preschool children with CSNHL. In addition, the insula, a key site within the auditory network, is primarily responsible for sound recognition and processing (61). Our previous study showed that local neuronal activity in the insular region of preschool children with CSNHL was abnormal, and functional network connectivity analysis revealed that the FC of the insula was significantly enhanced, suggesting that the insula may be involved in the process of functional reorganization of the auditory and visual cortices.

#### 4.2.2 FC changes in the proprioceptive cortex and motor cortex in preschool children with CSNHL

In this study, we found enhanced FC between the PAC and the bilateral precentral gyrus, postcentral gyrus (BA4) and left paracentral lobule (BA4) using seed-based FC analysis.

The precentral gyrus is the human primary motor cortex and is an important structure for the execution of voluntary movements (48, 62). One study demonstrated that infants learn language by learning the connection between sounds and the movements required for articulation (49). This finding implies that synchrony between the auditory cortex and the motor cortex may be a key factor in language development (23). For preschool children with CSNHL, without sound stimulation, the synchronization between the auditory cortex and the motor cortex does not occur, and the motor cortex needs other types of sensory stimulation for development. Thus, perceptual compensation may also occur in motor-related cortices such as the precentral gyrus. The postcentral gyrus is the human somatosensory and motor center, with extensive connections with deep nerve nuclei, and is mainly responsible for the regulation of movement and the maintenance of posture (63). Some studies have shown that SNHL patients exhibit changes in the postcentral gyrus, indicating that the somatosensory system provides perceptual compensation for hearing loss (64). The paracentral lobule belongs to BA1, 3 and 4, the anterior part of which belongs to the frontal lobe, and the posterior part belongs to the parietal lobe (65). The anterior part of the paracentral lobule is the auxiliary motor area, and the posterior part is responsible for controlling the motor and sensory nerve function of the contralateral distal limb (63). Some studies have shown that children with severe SNHL exhibit significant changes in the auditory cortex and somatosensory and motor cortex but no significant changes in the visual cortex (66). The results of our FC analysis showed that the FC between the PAC and the bilateral precentral gyrus, postcentral gyrus and left paracentral lobule was enhanced in preschool children with CSNHL, suggesting that there was cortical reorganization or compensation between the visual cortex and the somatosensory and sensory cortex after auditory deprivation in deaf children. In our previous study, we revealed local functional changes in the somatosensory cortex in deaf children, which indicated that local functional changes could lead to whole-brain functional changes.

#### 4.2.3 Changes in FC in the salience network and default recognition network in preschool children with CSNHL

In this study, seed-based FC analysis also revealed significantly increased FC in the dorsal anterior cingulate gyrus, bilateral insula, and left inferior parietal lobule in preschool children with CSNHL.

The main hubs of the salience network (SN) (67) are located in the dorsal anterior cingulate cortex and bilateral insula, and they can integrate internal and external stimuli (68), including stimuli from the auditory pathway (69). The findings of a meta-analysis suggested that the SN plays an important role in switching between attention and cognitive resources (70). Shanshan Wang (34) reported that the FC of the anterior cingulate gyrus in the SN of patients with SNHL was enhanced. The insula is mainly responsible for sound recognition and processing (61). In this study, preschool children with CSNHL had increased FC in the dorsal anterior cingulate cortex and insular lobe, which may be related to abnormal monitoring and processing of information from themselves or the outside world, indicating that preschool children with CSNHL may be highly responsive to environmental changes or external stimuli after auditory deprivation.

The DMN (71, 72) includes areas of the brain that are activated when the brain is at rest and individuals are not engaged in goal-directed activity. The DMN is involved in self-reference, social cognition, episodic and autobiographical memory, and linguistic and semantic memory (73). The existence of the DMN is universal (72). The DMN is known to exist in newborn infants (74, 75), but the internal connection strength is not as strong as that in adults (76). Some researchers (77, 78) have suggested that enhanced DMN activity may be related to psychological activities such as self-judgment, episodic memory and social cognition, while the activity is weakened when individuals participate in external attention tasks. Our study showed that the FC of the inferior parietal lobule in the DMN of children with SNHL was enhanced, suggesting that this increase may be related to the increase in self-information processing and attention after hearing loss in children, which is consistent with previous reports. However, some studies have shown that the FC of the inferior parietal lobule in the DMN of SNHL patients is weakened (79), which is not consistent with the findings of this study and may indicate that the course of auditory deprivation and the different reorganization patterns of the DMN in children with CSNHL are related.

### 4.3 Changes in the mean ALFF, ReHo and FC of the thalamus in preschool children with CSNHL

The thalamus is considered the highest center of sensation through which a large amount of information enters the cerebral cortex (80). Therefore, thalamic nuclei are essential for connecting the cortex and subcortex and coordinating cortical and subcortical FC (81). It has been shown (82, 83) that the medial genu and lateral genu within the thalamic nuclei are relay stations for auditory and visual information, respectively; for example, the medial genu transmits auditory information from the inferior colliculus to the auditory cortex. However, there is also evidence that the medial nerve plays a broader role in multisensory processing (84). In this study, the ALFF and ReHo in the thalamus were significantly increased, and the FC between the PAC and thalamus was enhanced, suggesting that after auditory deprivation in preschool children with CSNHL, the activity of motor neurons in other sensory cortices (such as visual, auditory, and proprioceptive cortices) increased, and these neurons projected to the center of the visual cortex through the thalamus. Furthermore, auditory deprivation affects the auditory cortex, which leads to cortical reorganization or functional compensation.

### 4.4 Effect of age on the development of brain function in preschool children with CSNHL

During brain development, there is a period of maximum neuroplasticity in the development of auditory center, which is called “critical period of developmental plasticity” (85). The lack of auditory input has a serious impact on brain function during this critical period, especially the auditory and visual cortex happens cross modal restructuring and repositioning cortex function (13). Studies have shown that the critical period of developmental plasticity of auditory cortex exists between birth and 2–4 years old, and CI performed during this period is also more likely to obtain the best rehabilitation effect (34). An fMRI study of children with SNHL before CI showed that the PAC had enhanced FC between brain regions involved in auditory and language networks in the SNHL group, which may reflect functional compensatory reorganization of the cerebral cortex after hearing loss (34). This suggests that age plays a key role in the development of brain function in deaf children after auditory deprivation. In our study, the average age of the subjects was about 3 years old, and the auditory cortex underwent cortical functional reorganization with other cortices. And correlation analysis showed that that there was a weak negative correlation between the ALFF and FC values involving the auditory cortex, visual cortex and sensorimotor cortex and CAP score, suggesting that CI implantation in the sensitive period of plasticity may use the recovery of auditory and language after CI, and exceeding the sensitive period may be detrimental to the recovery of auditory and language after CI.

### 4.5 Limitations and future directions

The present study has several limitations: (1) the participants were preschool children with severe or very severe SNHL, and the sample size was small, especially that of the HCs, which may have influenced the results. In the future, the sample size needs to be expanded, and the relationship between brain function changes and the course of disease in preschool children with CSNHL needs to be further studied. (2) No analysis based on different age groups was performed. In future studies, the sample size will be expanded to conduct research in this area. (3) The CAP score was determined only at 6 months after CI in preschool children with CSNHL. At this time, some children may not have adapted to the cochlear implant, so long-term follow-up of CAP scores is needed to verify the relationship between brain function changes and clinical scores.

## 5 Conclusion

In summary, this study investigated the brain function of preschool children with CSNHL through ALFF, ReHo and SEED-based FC analyses. There are local changes in ALFF and ReHo in auditory cortex, visual cortex, PFC and somatic motor cortex after auditory deprivation in preschool children with CSNHL, which suggests that the cortical function of these regions has changed. In this process, the PFC may play a top-down regulatory role. Further FC analysis using the PAC as the seed showed that the FC between the PAC and the visual cortex and between the proprioceptive cortex and motor cortex was enhanced after auditory deprivation in preschool children with CSNHL, suggesting that cortical function reorganization or compensation occurred between hearing and these cortex in children with CSNHL after auditory deprivation. Moreover, the functional reorganization or compensation of these cortex may not be conducive to the recovery of auditory language after CI.

## Data availability statement

The datasets presented in this article are not readily available because requests must be approved by the Ethics Committee of The Affiliated Hospital of Guizhou Medical University. Requests to access the datasets should be directed to the first author YY, yinyi_gzgy@163.com.

## Ethics statement

The studies involving humans were approved by the Ethics Committee of Affiliated Hospital of Guizhou Medical University. The studies were conducted in accordance with the local legislation and institutional requirements. Written informed consent for participation in this study was provided by the participants’ legal guardians/next of kin.

## Author contributions

YY: Methodology, Project administration, Software, Visualization, Writing – original draft, Writing – review & editing, Data curation, Formal analysis. XL: Data curation, Investigation, Writing – review & editing. JZ: Writing – review & editing, Methodology, Software. KY: Writing – review & editing, Methodology, Data curation. MH: Formal analysis, Writing – review & editing, Methodology. GS: Writing – review & editing, Supervision, Validation. CH: Software, Writing – review & editing. ZW: Writing – review & editing, Software. HY: Supervision, Writing – review & editing, Formal analysis, Validation. BG: Writing – review & editing, Formal analysis, Supervision, Validation.

## Footnotes

^1^ http://www.Fil.ion.ucl.ac.uk/spm/software/spm12/

^2^ http://rfmri.org/dpabi

^3^ http://www.fil.ion.ucl.ac.uk
